# Development of a food product profile for boiled and steamed sweetpotato in Uganda for effective breeding

**DOI:** 10.1111/ijfs.14792

**Published:** 2020-10-08

**Authors:** Robert O. M. Mwanga, Sarah Mayanja, Jolien Swanckaert, Mariam Nakitto, Thomas zum Felde, Wolfgang Grüneberg, Netsayi Mudege, Mukani Moyo, Linly Banda, Samuel Edgar Tinyiro, Sarah Kisakye, David Bamwirire, Beatrice Anena, Alexandre Bouniol, Damalie Babirye Magala, Benard Yada, Edward Carey, Maria Andrade, Suzanne D. Johanningsmeier, Lora Forsythe, Geneviève Fliedel, Tawanda Muzhingi

**Affiliations:** ^1^ International Potato Center Ntinda II Road, Plot 47, Naguru Hill, Box 22274 Kampala Uganda; ^2^ International Potato Center Apartado 1558 Lima Peru; ^3^ International Potato Center Box 25171 Nairobi Kenya; ^4^ Department of Molecular Biology and Biotechnology Pan African University Institute of Basic Science, Technology and Innovation JKUAT P.O Box 62000 00200 Nairobi Kenya; ^5^ National Agricultural Research Laboratories P. O Box 7065 Kampala Uganda; ^6^ Faculté des Sciences Agronomiques Université d’Abomey‐Calavi 01 BP 526 Cotonou Benin; ^7^ Qualisud, Univ Montpellier, CIRAD, Montpellier SupAgro, Univ d'Avignon, Univ de La Réunion Montpellier 34 398 France; ^8^ National Agricultural Research Organisation‐Mukono Zonal Agricultural Research and Development Institute P.O. Box 164 Mukono Uganda; ^9^ National Agricultural Research Organization National Crops Resources Research Institute Namulonge, P.O. Box 7084 Kampala Uganda; ^10^ International Potato Center C/O Crops Research Institute P.O. Box 3785 Kumasi Ghana; ^11^ International Potato Center IIAM Av. FPLM 2698, P.O. Box 2100 Maputo Mozambique; ^12^ United States Department of Agriculture Agricultural Research Service Southeast Area Food Science and Market Quality & Handling Research Unit 322E Schaub Hall Raleigh NC 27695 USA; ^13^ Natural Resources Institute University of Greenwich Chatham ME4 4TB UK; ^14^ CIRAD UMR QUALISUD Montpellier F‐34398 France

**Keywords:** Boiled roots, preferences, processing, product profile, quality characteristics, raw roots, sweetpotato

## Abstract

This study sought to understand user preferences of raw, boiled and steamed sweetpotato, a staple food in Uganda. A sequential methodology involving state of knowledge review, gendered food mapping, processing diagnosis and consumer testing was used in Lira and Kamwenge districts. Preferred raw sweetpotato characteristics were large roots (≥ 3 cm diameter) with a sweet taste, smooth skin and hard texture, while mealiness, sweet taste and good sweetpotato smell were important attributes for boiled sweetpotato. Processors, mostly women, highlighted ease of peeling and sappiness of raw roots. There were gender differences in quality characteristic preferences and perceived importance. The released variety, NASPOT 8, had the highest overall liking in Kamwenge and was well liked in Lira. Penalty analysis of consumer data showed that sweetness and firmness were key drivers of overall liking. The results will support breeding programmes in meeting specific end‐user product profiles, selection criteria and uptake of new varieties.

## Graphical abstract

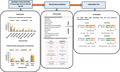

## Introduction

Sweetpotato (*Ipomoea batatas* L. Lam) is an important food and income security crop in Uganda (Gibson, 2013). The crop increased in importance following major threats to banana (banana bacterial wilt) and cassava (cassava mosaic virus) that ravaged productivity of these staples. However, after Uganda holding position as highest producer in Africa for several years, production plummeted from 2800 MT in 2011 to 1500 MT in 2017 (FAOSTAT, 2018), due to expansion of upland rice cultivation (Kankwatsa *et al*., 2019) and declining yields attributed to pests and diseases (Wokorach *et al*, 2019). Nonetheless, sweetpotato remains a food staple (Mwanga & Ssemakula, 2011), which bridges hunger gaps (Namanda *et al*., 2011). Sweetpotato is consumed after steaming, boiling, frying or roasting (Odora *et al*., 2000). Other products include puree, dried chips and chunks, flour, pastries and confectionery (Nakanyike, 2014; Abong *et al*., 2016; Bocher *et al*., 2017). Per capita annual consumption is estimated at 95 kg (Abong *et al*., 2016) with perceived increased demand (Kyalo *et al*., 2014; Okonya & Kroschel, 2014 ).

Farmers produce white or yellow‐fleshed varieties such as Dimbuka, Sukali, Tanzania, Kawogo, New Kawogo, Bwanjule, Sowola, Wagabolige and the Namulonge improved sweetpotato (NASPOT) series. Sweetpotato is grown biannually, mainly by women (Okonya & Kroschel, 2014). Households grow sweetpotato on shared plots, but roles and responsibilities are demarcated by gender as are crop decisions, influencing the choice of varieties grown (Mudege *et al*., 2016). Gilligan *et al*. (2014) noted that while men decide on overall crop choices, women actively engage in crops and varietal selection for consumption. Evidence indicates that women play an important role in the adoption of new sweetpotato varieties, but this is a joint spousal decision (de Brauw *et al*., 2012; Gilligan *et al*., 2014). Women also play a strong role in preparing boiled or steamed sweetpotato. For these reasons, consulting both women and men and disaggregating preferences for quality characteristics by gender is important.

In sub‐Saharan Africa (SSA), major advances in breeding sweetpotato have led to the release of superior productive and nutritious varieties (Shumbusha *et al*., 2014; Ssemakula *et al*., 2014; Andrade *et al*., 2016; Mwanga *et al*., 2017). Contemporary breeding programmes focus mostly on agronomy‐related characteristics and less on end‐user preferences, resulting in slow adoption of improved varieties (Jenkins *et al*., 2018). Sweetpotato food chains are characterised by diverse actors with varying varietal choice preferences, attributed to factors such as socio‐economic and gender dynamics (Mudege & Grant, 2017; Weltzien *et al*., 2020). Key preferences guiding varietal selection and market characteristics of raw sweetpotato include good taste (sweet), high dry matter content associated with hard texture, low fibre content, red and cream skin colour, large roots, white flesh colour, oval shape, cleanliness and absence of disease symptoms such as spots (Kilimo Trust, 2013). However, preferences for quality characteristics by the type of user or gender have not been analysed and physical and chemical characteristics need to be defined for breeders to address user needs so as to increase adoption of new varieties. Sensory preferences of African consumers have been deficient, focusing on physicochemical and nutritional profiling, safety and cost of products (Rakotosamimanana & De Kock, 2020). Additionally, several gaps were identified in recent consumer studies such as inadequate detail in methodology and use of test subjects that were not representative of the target consumers. Sensory studies are significant because one of the major determinants of food acceptability is consumer satisfaction derived from specific sensory quality attributes (Costell *et al*., 2010).

This gendered study was undertaken to assess preferred characteristics of sweetpotato for steaming and boiling by different end‐users in northern and western Uganda. The study findings will inform integration of user preferences and needs in the East African sweetpotato breeding programmes and define a new food product profile, thus guiding the selection criteria for sweetpotato breeding.

## Materials and methods

The study was conducted in Lira in northern Uganda and Kamwenge in western Uganda between September 2018 and December 2019 as part of a project‐wide study. Lira and Kamwenge are major producers of sweetpotato for food and income, and four communities in each of these districts were selected for the study.

### Identification of quality characteristics of raw and boiled/steamed sweetpotato

The study used a mixed‐methods research design based on an interdisciplinary and participatory methodology (Forsythe *et al*., 2020). This included conducting a state of knowledge (SOK) review, key informant interviews (KIIs), sex‐disaggregated focus group discussions (FGDs), individual and market interviews (IIs and MIs), food preparation, and consumer tests (Forsythe *et al*., 2018; Fliedel *et al*., 2018a; Fliedel *et al*., 2018b).

Gendered food mapping was first conducted in eight study communities in Lira and Kamwenge. In the first phase, a total of eight KIIs (five female, three male); 16 FGDs (eight male, eight female) involving 128 respondents (Sixty four men, Sixty four female); 72 IIs (Sixty women, twelve men); and seven MI (five women, two men) were conducted . From these data, the first iteration of the product profile was developed detailing preferred and non‐preferred quality characteristics and varieties for boiled sweetpotato.

In the second phase, preparation diagnostics were conducted with eight women in Lira and Kamwenge districts to evaluate the boiling and steaming of different selected sweetpotato varieties and the desired quality characteristics. These included: liked and disliked characteristics at each preparation step up to consumption, changes in root yield after peeling, duration of each preparation step and boiling/steaming temperature. Kiribwamukwe, Otandibata, and Ndererabana varieties were used for the study in Kamwenge while in Lira, they were Okonynedo, Arakaraka and Otada. NASPOT 8, an improved variety commonly grown in Lira and Kamwenge, was included as a control in both districts. These varieties were also used for the consumer tests.

Consumer tests, including hedonic ratings and just about right (JAR) scores (Fliedel *et al*., 2018b), were conducted in the rural and town areas in Lira and Kamwenge districts, with 246 consumers (100 male and 146 female). A nine‐point hedonic scale ranging from extremely dislike (1) to extremely like (9) was used to assess the overall liking of the four boiled/steamed sweetpotato products in each district. A three‐point JAR scale (not enough, just about right and too much) was used to assess four quality characteristics (colour, sweetness, firmness and mealiness) identified as important attributes during the previous surveys (FGDs and IIs) and preparation diagnosis with processors.

Boiled or steamed sweetpotatoes for consumer testing were prepared under hygienic conditions (Fliedel *et al*., 2018b). For boiled sweetpotato, each sample of raw sweetpotato was peeled, washed twice and boiled separately in a saucepan. Steamed sweetpotato was prepared in a similar manner, but each sample was wrapped in banana leaves before steaming. Each sample was assigned a code so that participants would not know which variety they were evaluating. Markers (such as banana fibres) were used to identify each coded sample. The coded samples (≈40 g each) were served individually in a randomly assigned order following a balanced complete block design. Serving temperatures were maintained at 80 ^0^C through use of insulated dishes.

Participants in KIIs, FGDs and preparation diagnosis were selected with the aid of World Vision and Samaritan’s Purse field staff. The selection criteria were: being actively involved in production, preparation and consumption of sweetpotato. Participants in the IIs and MIs and consumer test were randomly selected using systematic random sampling using an interval of three. However, for the IIs, there was sample disproportionality which could be attributed to women being more heavily involved in production and preparation of boiled/steamed sweetpotato. In all cases, only adults aged 18 and above were included in the study. All the participants were regular consumers of boiled/steamed sweetpotato.

### Ethical considerations

Participants were fully informed about the study, and they could stop the interview at any step. The research respected the rules of voluntary participation and anonymity. Informed consent was received from all participants before the study. The study was approved by the Institutional Review Board of the Uganda National Council of Science and Technology, Kampala, Uganda (reference, MAKSSREC 12.19.364).

### Data analysis

Qualitative data were coded and analysed as described by Forsythe *et al*. (2018). In order to obtain priority rankings for most preferred and least preferred varieties and characteristics from FGDs, IIs and MIs. Rankings of importance were aggregated, and weights applied to show averages by gender, region and for the total respondents.

Quantitative data from preparation diagnosis were analysed using SPSS version 22 (IBM, Chicago, IL, USA). Yield and duration of each preparation step and cooking temperatures were compared in generalised linear models with regions as a covariate (ANCOVA). Consumer data were analysed in XL STAT (2017 Addinsoft Inc, 244 Fifth Avenue, Suite E100 New York, N.Y. 10001) using descriptive statistics for the JAR test. Hedonic data were analysed by ANOVA to compare overall liking of the test varieties, and multiple comparisons were used to separate the means (Tukey’s test). Differences were considered significant in cases where *P* < 0.05.

Penalty analysis of consumer data was done using JMP Pro 14 (SAS Institute, Cary, NC, USA). Overall liking scores were modelled with Colour JAR, Sweetness JAR, Firmness JAR and Mealiness JAR as categorical variables using an ANOVA (Pages *et al*., 2014). Means separation and statistical significance of differences between means were achieved with a Tukey’s test or simple t‐test as appropriate. A penalty of> 1 observed for at least 20 % of consumers was used as criteria to determine the product attributes that are targeted for further product improvement.

## Results

### Main varieties used for boiled sweetpotato in Lira and Kamwenge districts

Results from II’s revealed variations in preferences between men and women, and between respondents from Lira and Kamwenge districts. Women preferred Okonynedo, NASPOT8 (= NASPOT 8) and Arakaraka (in descending order) while men preferred NASPOT8, Kakamega and Okonynedo. Okonynedo was chosen because of its high yield (50% of respondents), early maturity (30%) and sweet taste (20%). Men also liked this variety because it did not rot easily. NASPOT8 was chosen because of its high yield as reported by 60% women and 80% men. This difference, along with the difference in ranking of the variety between men and women, likely indicates that men place more importance on high yield compared to women. Men further appreciated its early maturity and large roots while women liked its sweet taste. All male respondents indicated that they grew the same varieties as their spouses; however, over half (58%) of female respondents indicated that they grew the same varieties as their spouses. In Kamwenge, the most preferred variety was Kyinzali, an orange/yellow‐fleshed local landrace. This was followed by the orange‐fleshed sweetpotato (OFSP) variety, NASPOT8. Respondents who did not recall the names of improved OFSP varieties referred to them as *OFSP*. In Lira, Okonynedo, Arakaraka and NASPOT8 were preferred (in descending order). Preferences were skewed towards OFSP because many respondents had been sensitised about the nutritional aspects of these varieties. Seventy‐five per cent of respondents in Kamwenge and 58% in Lira liked NASPOT8 because it was nutritious. In Lira, the local variety, Arakaraka, was preferred because of its early maturity, high yield and ease of peeling.

From FGDs (Table 1), men ranked NASPOT8, Kiribwamukwe, Okonynedo and NASPOT13O (= NASPOT 13 O) as the most important varieties, which slightly differed from II findings. Women mentioned Okonynedo, NASPOT8 and Arakaraka, consistent with II findings. Interestingly, men did not mention Arakaraka variety among the three important varieties in the IIs nor FGDs. At the regional level, Kamwenge respondents preferred NASPOT8, Vita, Kiribwamukwe and NASPOT13O, while Lira reported Okonynedo, Arakaraka and NASPOT 8 as the most desired varieties (in descending order), which was similar to the results from the IIs. Both regions rated NASPOT 8 highly due to its agronomic (early maturity, high yield) and sensory characteristics (large root, sweet taste) which were like their local varieties. In Kamwenge, Kiribwamukwe was preferred because of its sweet taste and perceived to be superior to improved OFSP varieties.

**Table 1:** Improved (I) and local (L) sweetpotato varieties mentioned in focus group discussions (FGDs) in order of priority by gender and region

Men’s FGD	Women’s FGD	Kamwenge FGD	Lira FGD
1. NASPOT 8 (I)	1. Okonynedo (L)	1. NASPOT 8 (I)	1. Okonynedo (L)
2. Kiribwamukwe (L)	2. NASPOT 8 (I)	2. Vita (I)	2. Arakaraka (L)
3. Okonynedo (L)	3. Arakaraka (L)	2. Kiribwamukwe (L)	3. NASPOT 8 (I)
3. NASPOT 13O (I)	4. Apakapaka (L)	2. NASPOT 13O (I)	4. Apakapaka (L)
5. Awitongweno (L)	4. OFSP‐UK* (I)	5. Kabode (L)	5. Liralira/Otada (L)
5. Kabode (I)	4. Vita (I)	6. OFSP‐UK* (I)	6. Awitongweno (L)
5. OFSP‐UK* (I)			6. OFSP‐UK* (I)
5. Vita (I)			

* OFSP^‐^UK – unknown OFSP varieties.

Traders mostly preferred local varieties because of their marketability. In Lira, Otada, Okonynedo and NASPOT8 varieties were preferred, while Kamwenge traders preferred Kyinzali followed by Matamabuk, Lwentuuma and NASPOT8.

### Less preferred sweetpotato varieties

Women and men FGD respondents in Kamwenge had a lower preference for the released varieties, Vita and Kabode, because of low yields, slow growth and poor sensory characteristics (softness, small size and fibrousness), resulting in low marketability. Least preferred local varieties were Ejumula, Ndererabaana, Kakazikamalyo and Kahogo. However, these varieties were still grown because of scarcity of planting material of preferred varieties and food security. Other less preferred varieties were the local landraces, Rushema mahamba and Rwamityana which were no longer grown. In Lira, men mentioned Agoba, Peninah, Anamoito, Oleke and Otede while women highlighted Dwelacel among the less preferred varieties. Reasons for low preference included late maturity, low yield, fibrousness, softness, mild sweetness and short shelf life. All these varieties except Agoba were no longer cultivated in Lira. Generally, respondents in both regions rated the local varieties superior for both sensorial and agronomic‐related characteristics compared to most released improved varieties like Vita and Kabode.

### General quality characteristics of a desirable raw sweetpotato – perceptions of farmers and traders

Overall, preference for sweetpotato was mainly associated with morphological (large roots and smooth skin) and physicochemical (sweet taste and hard root) characteristics. Preferences for specific quality characteristics were similar by gender and region in the IIs, that is large, hard root and smooth skin. However, there were gender differences as women ranked sweetness first while men ranked it fifth. Kamwenge and Lira respondents concurred on large root and sweet taste as priority characteristics but differed on hard root (Kamwenge) and smooth skin (Lira). From the FGDs, the priority characteristics for men were large, hard root, no damage and roots which produce sap whereas the ones for women were large, hard root and smooth skin. Large and hard root were common for both men and women; as in the IIs. Between the regions, the top priority characteristics differed. In Kamwenge, the preferred characteristics were hard root, sap production when a raw root is cut and sweet taste. FGDs in Lira preferred large roots, smooth skin and no physical or insect damage. Traders highlighted the following raw sweetpotato characteristics as important for a desirable boiled sweetpotato: large root, hard, smooth skin (not damaged) and moderately sweet.

### General poor‐quality characteristics of raw sweetpotato – farmer perceptions

The least preferred characteristics (from IIs) for women were watery, soft and small roots whereas the men considered rough skin, soft and fibrous roots as least preferred quality characteristics. Both men and women ranked soft root among the least preferred characteristics. However, watery was considered the least preferred raw sweetpotato characteristic, while it was fourth for men.

In Kamwenge, the least preferred characteristics were soft, small, fibrous and infected roots while in Lira, they were watery, rough skin and fibrous roots. Respondents from both regions did not like fibrousness.

### General quality characteristics of boiled sweetpotato – farmer perceptions

Nearly identical high‐quality characteristics for ready‐to‐eat boiled sweetpotato were cited by respondents during the FGDs and IIs: sweet taste, good smell, mealy, firm and non‐fibrous. According to IIs, both men and women ranked sweet taste, mealiness and good smell as priority characteristics. However, women specifically mentioned not watery and split surface as preferred characteristics while non‐fibrousness was unique to the men. There was considerable divergence for tastelessness – women ranked it first among poor characteristics (least preferred) of boiled sweetpotato while men ranked it fourth. In Kamwenge, respondents identified mealiness, firm and split surface of root while for Lira it was sweet taste, mealiness and good smell. Mealiness and sweet taste were the most differentiating characteristics by gender and region in the FGDs. Regarding the poor‐quality characteristics of boiled/steamed sweetpotato cited in FGDs and IIs, men mentioned softness, bad taste and bad smell in Kamwenge district, while women mentioned watery, tasteless, soft, fibrousness and bitter taste, in Lira district (Table 2).

**Table 2:** Perceptions of good‐ and poor‐quality sweetpotato characteristics by women and men respondents in individual interviews

Criteria	Good characteristics			Poor characteristics		
	Raw material	Processing	Boiled/steamed sweetpotato	Raw material	Processing	Boiled/steamed sweetpotato
Characteristics that were only mentioned by men	No damage to skin	Sappy, low water content, ease of cooking	Attractive colour	Rotten	Bad skin colour	Bad taste, bad smell, not sweet
Characteristics that were only mentioned by women	Sweet taste	Hard root, good appearance	NR	Small root, tasteless	Soft (when you break)	Watery, fibrous, bitterness
Ranked characteristics by men	1. Large root 2. Smooth skin 3. Hard root	1. Good smell 2. Sappy 3. Easy to peel	1. Sweet taste 2. Good smell 3. Mealy	1. Rough skin 2. Soft 3. Fibrous	1. Small root size 2. Pale skin colour 3.Presence of ridges and cracks	1. Soft 2. Bad taste 3. Bad smell
Ranked characteristics by women	1. Large root 2. Hard root 3. Smooth skin	1. Sweet taste 2. Good smell 3. Hard root	1. Sweet taste 2. Mealy 3. Good smell	1. Watery 2. Soft root 3. Small root	1. Small root size 2. Spotted flesh 3. Rough skin	1. Tasteless 2. Watery 3. Soft
Characteristics were ranked differently by men and women (1 = first; 5 = last)	Hard root (M = 3; W = 2) Smooth skin (M = 2; W = 3) Good smell (M = 4; W = 5)	Sweet taste (M = 5; W = 1) Good smell (M = 1; W = 2) Easy to peel (M = 3; W = 5)	Mealy (M = 3; W = 2) Good smell (M = 2; W = 3)	Watery (M = 4; W = 1) Fibrous (M = 3; W = 4)	Bad appearance (M = 3; W = 5) Rough skin (M = 4; W = 3)	Tasteless (M = 4; W = 1) Soft (M = 1; W = 3)

### Gender profiling

There were quality characteristics that were cited more often or only by men or women, and differences in the ranking of importance (Table 2). These gender differences were analysed for potential conflict or trade‐off for either sex, with regard to characteristics to prioritise for breeding objectives. While in some instances these are subtle differences, they are important. As breeders are only able to focus on a few traits at a time, knowing the most important characteristics for both men and women are vital in ensuring that released varieties have the 'must have' characteristics desired by their users. Furthermore, the evidence shows how preferences for certain varieties and characteristics are linked to gender roles in the food chain: women mentioned a number of characteristics important for ensuring a good, high‐quality product, related to their expertise in preparing boiled sweetpotato.

### Preparation diagnosis and quality characteristics – perceptions

**Figure 1:**
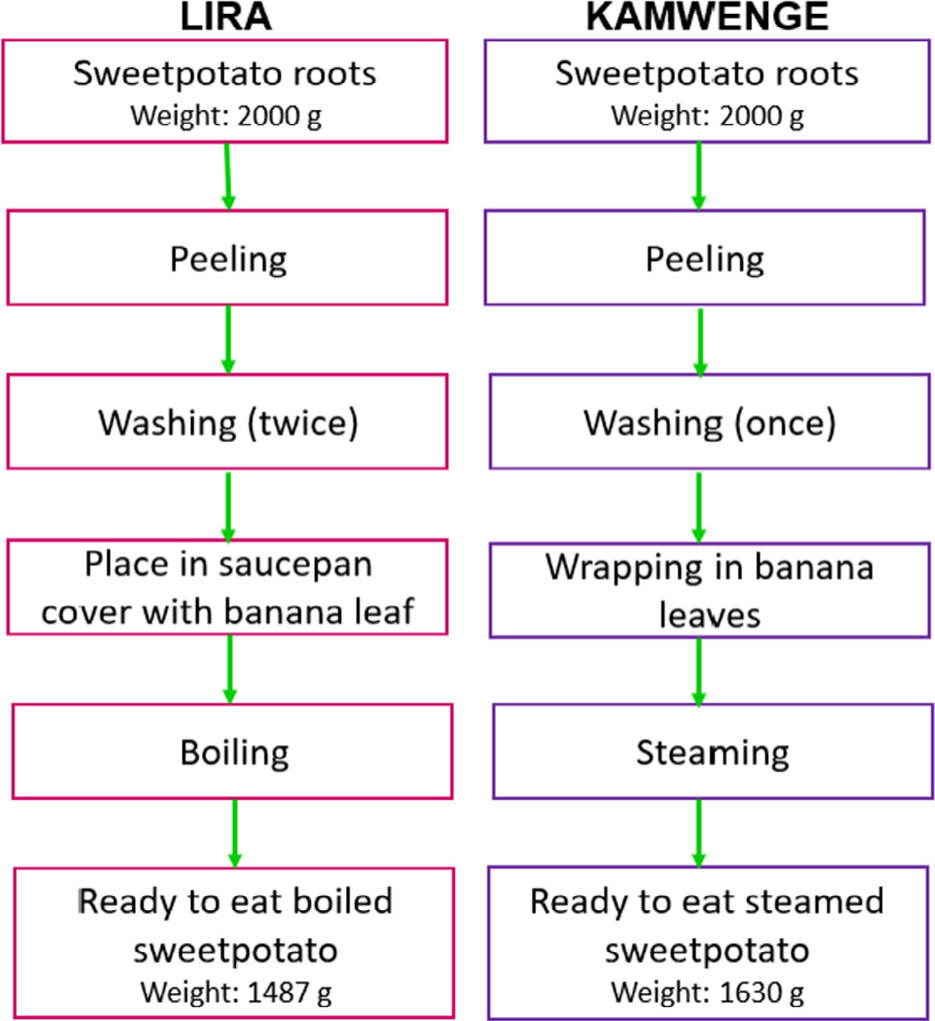
Flow diagram of boiling or steaming sweetpotato in Lira (left) and Kamwenge (right), Uganda.

The steps in boiling and steaming sweetpotato in Lira and Kamwenge, respectively, are shown in Fig 1. In Lira, the peeled sweetpotatoes were immersed directly in cold water and boiled in a saucepan covered with either banana leaf or another saucepan. Cooking time was measured from the time sweetpotatoes were placed on fire until when deemed ready. In Kamwenge, peeled sweetpotato roots were wrapped in banana leaves, placed in a saucepan with water, banana stalks and leaf midribs at the base of the saucepan and thereafter steamed. There were variations in yield after peeling, peeling and boiling duration among varieties (Table 3). In Kamwenge, NASPOT8, Kiribwamukwe and Otandibata had significantly higher post‐peeling yields than Ndererabana. The duration of peeling Kiribwamukwe was significantly shorter than that of Otandibata and NASPOT8. Preparation parameters were not significantly different among varieties in Lira. Post‐peeling yield of NASPOT8 was not significantly different between the two districts. On average, steaming in Kamwenge used more water (1 562.9 mL vs. 656.2 mL), which might have contributed to a longer steaming time (63.9 min) compared to boiling time in Lira (46.8 min.

**Table 3:** Preparation parameters of boiled/steamed sweetpotato

Variety/ Location	Peeling time (min)	Post‐peeling yield (%)	Boiling time (min)
Lira			
Arakaraka	10.0 ± 1.1a	80.5 ± 5.0a	42.4 ± 14.5a
Otada	7.7 ± 1.4a	77.4 ± 5.7a	43.4 ± 6.8a
NASPOT 8	8.4 ± 2.4a	77.8 ± 1.8a	52.6 ± 2.5a
Okonynedo	10.3 ± 2.5a	82.1 ± 8.6a	48.8 ± 9.8a
Kamwenge			
Kiribwamukwe	6.4 ± 0.6b	82.2 ± 1.2a	65.7 ± 6.7a
NASPOT 8	9.4 ± 1.3a	83.6 ± 3.6a	63.1 ± 7.1a
Ndererabana	8.1 ± 0.4ab	71.8 ± 3.5b	68.4 ± 12.1a
Otandibata	10.1 ± 1.5a	81.4 ± 3.6a	61.7 ± 13.3a

Different letter denotes significant difference between means (*P *< 0.05).

In each district, the processors reviewed the quality characteristics of the selected raw sweetpotato varieties before and during preparation. In Lira, the characteristics of good raw sweetpotato were smooth skin, big roots and white flesh colour, while in Kamwenge, they were sappy, homogenous red skin colour, no spots, hard root and firm skin. The least liked characteristics in Lira were rough skin, a lot of sap, dark spots and soft or watery roots, while in Kamwenge they were non‐uniform or pale skin colour, skin with black spots and fibrous roots.

During peeling, in both Kamwenge and Lira, indicators of a high‐quality boiled/steamed product were indicated as: firm, large roots with white‐, yellow‐ or orange‐coloured flesh and easy to peel. In Lira, varieties with moderate sap were preferred while in Kamwenge varieties with a lot of sap were preferred. In both regions, production of sap during peeling was perceived as an indicator of freshness. In Lira, roots with too much sap were difficult to wash and the residual sap could be detected in the boiled roots, which was undesirable.

There were variations between the regions in the quality characteristics described during washing. In Lira, the following were considered; smooth surface and no sap/gum when washing and the importance of washing twice to remove soil and sap as residual sap would affect the appearance (black marks) and taste (sappy/astringent taste). Washing was considered complete when all sap was removed, and the roots were not sticky/gummy. In Kamwenge, the following were considered, good‐quality characteristics such as ‘no eyes’/black spots, colour retention (white/yellow) and ‘not slippery’ (has starch) when washing. Colour change from white or yellow to brown or black (due to oxidation) was considered an important poor‐quality characteristic when washing.

For boiling in Lira, quality characteristics such as sweet smell of boiling sweetpotato, soft to touch (surface) and development of whitish/floury spots and sometimes cracks on the surface when ready were indicative of a preferred variety. Sweetpotatoes were deemed ready when all boiling water had evaporated. Estimating the correct amount of water to prevent undercooking or burning or overcooking which resulted in watery and tasteless roots was important. These characteristics were similar in both regions. In Kamwenge, an additional indicator for readiness was change in colour and softness of the banana leaves used for wrapping.

After preparation, the boiled/steamed sweetpotato varieties in Lira and Kamwenge were ranked by those that steamed (cooked) them. The top‐ranked variety in Lira was Otada followed by Okonynedo, NASPOT8 and Arakaraka. In Kamwenge, it was NASPOT8 followed by Kiribwamukwe, while Ndererabana was the least preferred. Characteristics of boiled/steamed sweetpotato as assessed by the cooks are summarised in Table 4. In both regions, whitish patches on the boiled/steamed sweetpotatoes indicated mealiness and thus deemed a good product. Mealy (powdery or crumbly like egg yolk), firm to the touch, not fibrous and not watery were preferred textural attributes in addition to sweet taste and characteristic sweetpotato smell in both districts.

**Table 4:** Good‐ and poor‐quality characteristics of boiled/steamed sweetpotato by district cited by processors at the end of processing

	Good‐quality characteristics		Poor‐quality characteristics	
	Lira	Kamwenge	Lira	Kamwenge
Appearance	Colour: white, yellow, orange, whitish patches	Colour: yellow, white, mealy (whitish patches on the inside) Shiny surface, smooth surface	Colour: black spots Cracked surface	Colour: pale, non‐uniform
Texture	Mealy (crumbly, powdery), soft when chewing, not watery, smooth in the mouth, firm to the touch, no fibres, smooth surface, thick	Mealy, firm to the touch, starchy (floury or powdery), not fibrous, dry (when touched and makes one thirsty when eating), sticky, not watery, long chewing time	Has fibres, watery, soft (mashy)	Fibrous, soft (mashy), watery, not mealy, short chewing time
Taste	Sweet taste	Sweet taste	Not sweet	Not sweet
Odour	Good smell	Good sweetpotato smell	No smell, off odour (fermented)	Off odour

### Consumer testing of boiled/steamed sweetpotato

**Figure 2:**
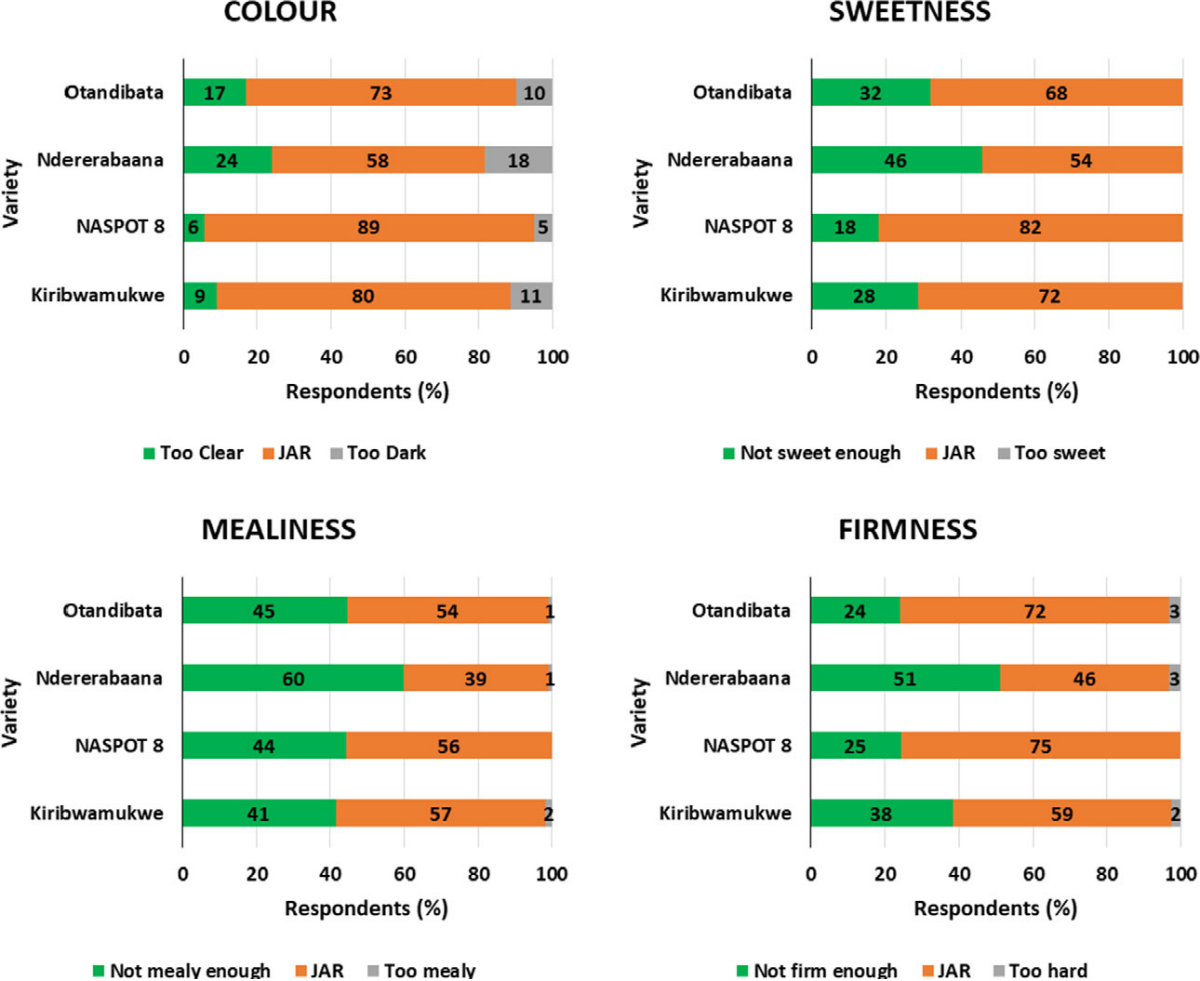
Just About Right (JAR) test conducted with consumers on four steamed final products made from different sweetpotato varieties grown in Kamwenge. Numbers inside bars show percentage of respondents for each rating. (*N *= 123).

**Figure 3:**
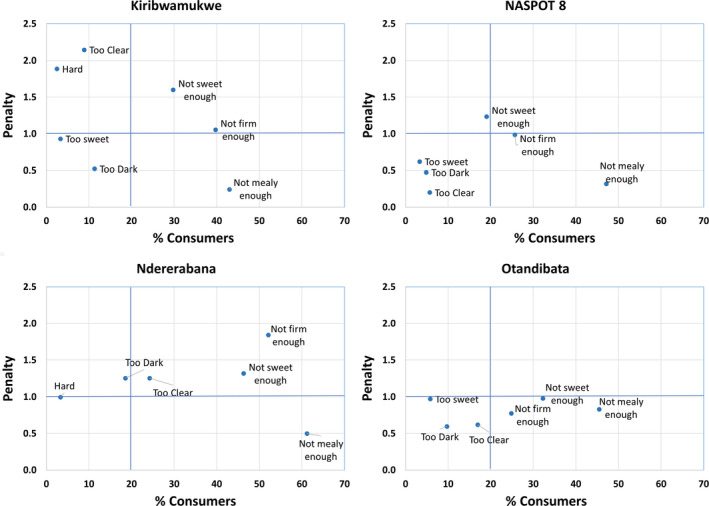
Penalty plots of the mean reduction in overall liking (penalty) vs. the percentage of consumers that classified each of the sweetpotato varieties in the Kamwenge consumer test as too little or too much of each product attribute.

Consumers were asked to taste one by one (rinsing in between) of the boiled/steamed roots prepared from four different varieties in each district. The mean overall liking of cooked sweetpotato varieties scored by consumers in Lira and Kamwenge districts is shown in Table 5. In Lira district, all four varieties tested were well liked and there was no significant difference in overall liking among the varieties. In Kamwenge district, there was a significant difference between NASPOT8 (most liked) and Ndererabana (least liked), and the intermediate varieties. Basing on the four characteristics identified for the JAR test (colour, sweetness, firmness and mealiness), more than 50% of consumers in Kamwenge scored all the characteristics 'Just about right' for steamed NASPOT8 sweetpotato (Fig. 2). Less than half of the consumers perceived that Ndererabana had the right intensity of mealiness (39%) or firmness (46%). Penalty analysis of varieties from Kamwenge is shown in Fig. 3 and Table 6. NASPOT8 was the most preferred variety overall; however, about 25% of consumers found it to be ‘not firm enough’ and scored it significantly lower in overall liking. Otandibata was penalised by a little over 30% of consumers that found it ‘not sweet enough’. Kiribwamukwe was penalised by consumers that found it both ‘not sweet enough’ and ‘not firm enough’. Ndererabana had the lowest overall liking and was penalised by ‘too clear’, ‘not sweet enough’ and ‘not firm enough’. Although a large proportion of the consumers found most varieties to be ‘not mealy enough’, the lack of desired mealiness had little to no effect on overall liking.

**Table 5:** Overall liking of boiled/steamed sweetpotato made from different varieties (with 246 consumers)

Origin of variety	Variety	Mean overall liking
Lira	Arakaraka	6.8^a^
	NASPOT 8	7.1^a^
	Okonynedo	6.5^a^
	Otada	6.7^a^
Kamwenge	Kiribwamukwe	6.6^b^
	NASPOT 8	7.4^a^
	Ndererabana	5.5^c^
	Otandibata	6.5^b^

Different letter denotes significant difference between means (*P* < 0.05). Hedonic scale (9 = Like extremely, 1 = Dislike extremely).

**Table 6:** Penalty analysis for sweetpotato varieties evaluated by consumers in Kamwenge and Lira districts of Uganda

	Sweetpotato variety		Colour		Sweetness		Firmness		Mealiness	
			Too Clear	Too Dark	Not sweet enough	Too sweet	Not firm enough	Hard	Not mealy enough	Too mealy
Kamwenge	Kiribwamukwe	Penalty*	2.1 ± 0.5	0.5 ± 0.5	1.6 ± 0.4	0.9 ± 0.9	1.1 ± 0.4	1.9 ± 1.6	0.2 ± 0.3	−1.1 ± 2.0
		*P*‐value	0.0003	0.5403	<0.0001	0.5285	0.0131	0.4817	0.7616	0.8718
	NASPOT 8	Penalty	0.2 ± 0.6	0.5 ± 0.6	1.2 ± 0.4	0.6 ± 0.7	1.0 ± 0.3	N/A	0.3 ± 0.3	N/A
		*P*‐value	0.9301	0.7012	0.0019	0.6635	0.0037	N/A	0.2938	N/A
	Ndererabana	Penalty	1.3 ± 0.4	1.3 ± 0.5	1.3 ± 0.3	−0.1 ± 1.1	1.8 ± 0.5	1.0 ± 1.2	0.5 ± 0.5	3.0 ± 2.4
		*P*‐value	0.0143	0.0210	0.0006	0.9920	0.0015	0.6797	0.5997	0.4393
	Otandibata	Penalty	0.6 ± 0.5	0.6 ± 0.6	1.0 ± 0.4	1.0 ± 0.7	0.8 ± 0.4	−0.2 ± 0.9	0.8 ± 0.4	−1.4 ± 1.8
		*P*‐value	0.3763	0.5279	0.0396	0.3941	0.1711	0.9648	0.0892	0.7348
Lira	Arakaraka	Penalty	1.5 ± 0.4	1.3 ± 0.5	1.6 ± 0.3	2.9 ± 0.6	0.3 ± 0.4	N/A	0.4 ± 0.4	−1.2 ± 0.9
		*P*‐value	0.0007	0.0289	<0.0001	<0.0001	0.3438	N/A	0.5588	0.3425
	NASPOT 8	Penalty	0.4 ± 0.6	1.1 ± 0.4	1.4 ± 0.3	N/A	0.5 ± 0.4	3.0 ± 1.5	0.7 ± 0.4	N/A
		*P*‐value	0.7327	0.0143	<0.0001	N/A	0.4249	0.1145	0.0470	N/A
	Okonynedo	Penalty	0.9 ± 0.4	1.2 ± 0.5	0.8 ± 0.4	0.9 ± 0.6	0.7 ± 0.4	0.7 ± 1.0	0.5 ± 0.4	0.0 ± 1.1
		*P*‐value	0.0330	0.0513	0.1613	0.3281	0.2232	0.7684	0.3233	0.9999
	Otada	Penalty	1.3 ± 0.4	1.9 ± 0.5	0.2 ± 0.4	1.4 ± 0.5	1.2 ± 0.4	0.4 ± 0.8	0.2 ± 0.4	−0.3 ± 0.9
		*P*‐value	0.0030	0.0008	0.8861	0.0167	0.0091	0.8414	0.8136	0.9427

* Penalty is the mean difference in overall liking between groups that classified the sweetpotatoes as just about right ('as I like') and those that classified it as too little or too much of any given attribute. Values are presented as the mean difference in liking ± the standard error of that difference with associated *P*‐values in the row below each penalty.

**Figure 4:**
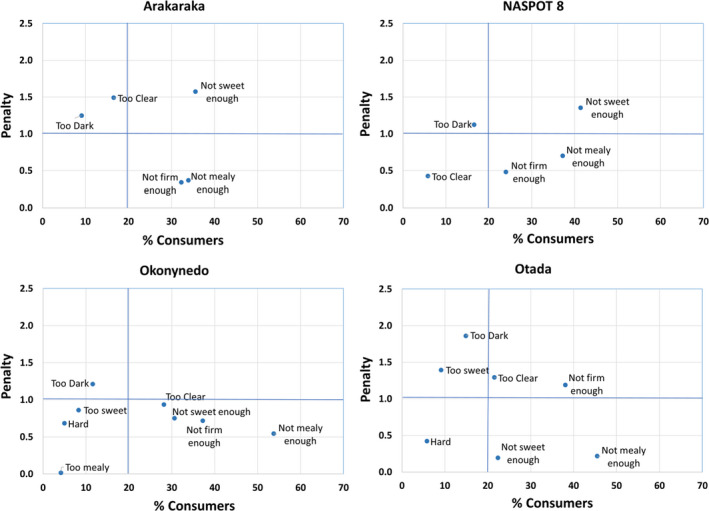
Penalty plots of the mean reduction in overall liking (penalty) vs. the percentage of consumers that classified each of the sweetpotato varieties in the Lira consumer test as too little or too much of each product attribute.

From Lira, NASPOT8 was scored JAR by 79% and 74% of consumers for colour and firmness, respectively, and was closely followed by Arakaraka with a JAR score of 75% and 68% for colour and firmness. Otada and Arakaraka were rated JAR by 76% and 64% of consumers for sweetness and mealiness, respectively. Okonynedo was scored JAR by only 42% of consumers for mealiness, and 54% of consumers found the boiled product not mealy enough. A lower percentage of consumers found JAR the sweetness of NASPOT8 and the firmness of Otada, 58 and 54 %, respectively. Penalty analysis of the varieties from Lira shows that Arakaraka and NASPOT8 were penalised by ‘not sweet enough’ (Fig. 4 and Table 6). Okonynedo was not penalised by any of the four sensory attributes. Otada was penalised by ‘too clear’ and ‘not firm enough’.

## Discussion

Large roots, sweet taste, hard root and smooth skin were preferred raw sweetpotato characteristics for both men and women and in both Kamwenge and Lira districts. Root size has been reported to be a good maturity index used by sweetpotato farmers in Uganda to inform the decision to harvest (Bashaasha *et al*., 1995). In addition, sweet taste and hard texture of the raw root are previously reported as highly desirable quality characteristics (Kilimo Trust, 2013). In this study, sweetness was highly prioritised by women but not men. Men indicated that they prefer ‘medium’ sweetness, hence the need for a trade‐off between the two. Raw and boiled sweetpotato sweetness has been found to vary considerably depending on factors such as variety, growing and storage conditions (Mwanga *et al*., 2009; Nakanyike, 2014). However, there seems to be no correlation of sweetness between fresh and boiled sweetpotato. Sweetness levels of some raw sweetpotato varieties in Ghana, observed as low or non‐sweet, changed to moderate sweet when cooked (Owusu‐Mensah *et al*., 2016). Sweet taste in raw sweetpotato is associated with glucose, fructose and sucrose while for boiled roots, it is attributed to maltose from starch hydrolysis by amylases during cooking (Lebot, 2017). Similarly, sweet potato like flavour of boiled sweet potato is positively correlated with maltose content (Laurie *et al*., 2013). The relationship of the raw root quality characteristics to the cooked root is very important, hence the need for studies to establish raw root characteristic predictors of the cooked root. Smooth skin was indicative of absence of disease symptoms and physical damage and therefore perceived as indicative of a good root. Small roots were not preferred by FGD and II respondents as they were deemed immature. Soft and watery roots were associated with disease and loss of freshness. Fibrous roots were also undesirable, similar to findings by Kilimo Trust (2013), where low fibre content was indicated among farmer preferences.

Sweet taste, mealiness and good sweetpotato smell were standout priority characteristics of boiled and steamed sweetpotato for men and women, and both regions. Our findings are in agreement with Tomlins *et al*. (2012), where preferred varieties had a characteristic sweetpotato flavour and crumbly (mealy) texture while those with watery texture were undesirable. A previous study in Uganda indicated that mealiness was an important varietal characteristic among farmers (Mwanga *et al*., 2007). Tumwegamire *et al*. (2011) also described desirable sweetpotato as being dry and starchy, while Mwanga *et al*. (2016) mentioned dry texture and sweet taste. Soft and watery boiled roots were associated with a lack of mealiness. Laurie *et al*. (2013) reported that wateriness was a disincentive for consumption of sweetpotatoes especially among SSA consumers who prefer roots with high dry matter. Bitter taste of boiled or steamed sweetpotato was singled out as undesirable by women. Sweetpotatoes produce phytochemicals such as coumarins and terpenoids when injured by insects (Mohanraj & Sivasankar, 2014) which could explain the bitter taste of some boiled or steamed roots. In a study of electric signals after application of chemicals on the tongue to ascertain the threshold for sweet, sour, salty and bitter tastants, women were reported to have lower thresholds for bitterness than men, further affirming the need for consideration of gender differences in taste perceptions in participatory breeding programmes (Wardwell *et al*., 2009; Gemousakakis *et al*., 2011).

The varietal differences in peeling time between Otandibata and Kiribwamukwe could be attributed to their peel and root texture. In most cases during steaming, Kiribwamukwe had a firm peel and root while Otandibata was said to have a soft peel and root. A soft texture means, preparation requires more care when peeling to conserve the flesh, hence a longer peeling duration. The differences in cooking time between Kamwenge and Lira districts were likely due to the differences in cooking methods (i.e. steaming and boiling). Other studies that used the two methods also reported a shorter cooking time for boiling than steaming (Preti *et al*., 2017). Food scientists should therefore carefully consider cooking time when developing laboratory‐based preparation methods for cooked sweetpotato samples for laboratory analysis.

Results from the consumer tests show that NASPOT8 from both Lira and Kamwenge was the most liked variety. This differs from previous studies which showed that consumers in SSA favoured white varieties over OFSP because of the sweetpotato flavour, smell and dry texture (Low *et al*., 2001; Tomlins *et al*., 2007, 2012; Laurie *et al*., 2013; Bowen *et al*., 2019). Previous studies have profiled OFSP varieties as having watery texture and pumpkin flavour with the white/pale ones being starchy, hard and coarse in texture with a sweet taste (Tomlins *et al*., 2007, 2012). However, breeding efforts have now bridged the dichotomous gap between flesh colour and associated sensory attributes (Low *et al*., 2001). This could explain consumer preference for NASPOT8, an OFSP variety with high dry matter content (32%), moderate sweetness and dry texture (Mwanga *et al*., 2009). Despite high overall liking, NASPOT8 was penalised by some consumers as ‘not sweet enough’ or ‘not firm enough’ in Lira and Kamwenge, respectively. Furthermore, Ndererabana, a local landrace variety from Kamwenge, had the lowest overall liking and was significantly penalised by a larger proportion of consumers as ‘not sweet enough’ and ‘not firm enough’. This augments Laurie *et al,* (2013) findings that wateriness and low dry matter content of a local cream‐fleshed sweetpotato variety contributed to its low acceptance. Characteristics such as hard texture and moderately sweet conform to consumer preferences especially among adults in SSA (Sugri *et al*., 2012). Although mealiness is a stated desirable attribute by sweetpotato consumers, a perceived lack of mealiness had little to no impact on overall liking for these varieties. In contrast, a lack of sweetness or firmness significantly decreased overall liking, suggesting that these sensory attributes can be targeted by breeders for developing improved varieties. The present study shows potential for increased adoption of improved varieties from participatory breeding, which is increasingly taking into account end‐user preferences. Similar findings from Mozambique show that the taste of OFSP varieties was equal or better than that of the white‐fleshed check cultivars (Andrade *et al*., 2016).

In this study, women’s appreciation of NASPOT8 for its high yield and taste characteristics can be attributed to their distinct role of ensuring household food security. For men, root size and good storage quality were the preferred characteristics, which could be linked to their role as major wholesalers in the food chain. Such preferences associated with gender norms are similar for cooking banana and cassava and are important in setting breeding goals and orienting gender needs, roles and responsibilities (Sanya *et al*., 2017; Teeken *et al*., 2018).

While some characteristics were easy to ascertain and define for profiling, such as large root, hard root and smooth skin, others like sweetness were not precise, with variations such as ‘moderate sweetness’ and ‘very sweet’. These may require further characterisation to define more accurately such characteristics in the boiled sweetpotato product profile. Breeding programmes therefore need to give critical attention to agronomic, processor and consumer‐related characteristics highlighted as key quality aspects for rapid varietal upscaling, adoption and end‐user acceptance. Raw and boiled characteristics of sweetpotato determining the preference by end‐users identified in this study will be translated into simple biophysical measurements and used to develop fast and accurate breeding procedures, high‐throughput phenotyping protocols, and in determining the genetics of major traits. Sweetpotato breeders working with food scientists, economists and gender experts across root, tuber and banana crops will be able to develop and rank the most important characteristics defined by food chain actors (producers, traders, processors and consumers) for the boiled sweetpotato product profile. The joint effort is expected to result in more efficient sweetpotato breeding in Uganda and East Africa, evidenced by inclusive product profile for boiled and steamed sweetpotato and resulting in improved varietal adoption in the East African region.

## Conclusions

This study identified the priority characteristics which sweetpotato users prefer for selecting roots for boiled or steamed sweetpotato, at various stages including selection of raw roots, preparation and at consumption. Large hard roots with sweet taste and smooth skin are preferred raw sweetpotato characteristics while sweet taste, mealiness and sweetpotato smell stood out for boiled and steamed sweetpotato across sexes and geographical locations. Preference of the orange‐fleshed NASPOT8 over the traditional white‐fleshed varieties in this study exhibits potential for increased adoption of nutritionally improved varieties that match local consumers’ sensory preferences. Surprisingly, mealiness was not a key driver of overall liking even though it is widely expressed as a desirable trait. However, sweetness and firmness are attributes that can be targeted for improvement by breeders to enhance consumer acceptance. Subtle differences were noted in gender profiling for desired characteristics such as sweetness being higher priority for women than men. Gender norms and roles influenced the characteristics men and women leaned to, thus calling for increased gender awareness in participatory breeding initiatives.

## Conflict of interest

The authors declare no conflicts of interests.

## Author contribution

**Robert Mwanga O.M. Mwanga:** Conceptualization (supporting); Methodology (supporting); Writing‐original draft (supporting); Writing‐review & editing (equal). **Sarah Mayanja:** Conceptualization (equal); Formal analysis (lead); Investigation (lead); Methodology (equal); Software (equal); Supervision (equal); Writing‐original draft (lead); Writing‐review & editing (equal). **Jolien Swanckaert:** Supervision (supporting); Writing‐review & editing (supporting). **Mariam Nakitto:** Formal analysis (supporting); Writing‐original draft (equal); Writing‐review & editing (supporting). **Thomas zum Felde:** Conceptualization (equal); Methodology (supporting); Writing‐review & editing (supporting). **Wolfgang Grüneberg:** Conceptualization (supporting); Writing‐review & editing (supporting). **Netsayi Mudege:** Conceptualization (equal); Funding acquisition (supporting); Supervision (supporting); Writing‐review & editing (supporting). **Mukani Moyo:** Conceptualization (equal); Supervision (supporting); Writing‐review & editing (supporting). **Linly Banda:** Writing‐review & editing (supporting). **Samuel Edgar Tinyiro:** Conceptualization (equal); Data curation (supporting); Formal analysis (equal); Investigation (equal); Methodology (equal); Software (equal); Writing‐original draft (equal); Writing‐review & editing (equal). **Sarah Kisakye:** Formal analysis (supporting); Investigation (supporting); Writing‐original draft (equal); Writing‐review & editing (supporting). **David Bamwirire:** Writing‐original draft (supporting); Writing‐review & editing (supporting). **Beatrice Anena:** Formal analysis (equal); Writing‐original draft (supporting); Writing‐review & editing (equal). **Alexandre Bouniol:** Methodology (supporting); Writing‐review & editing (supporting). **Damali Babirye Magala:** Writing‐original draft (supporting); Writing‐review & editing (supporting). **Benard Yada:** Writing‐review & editing (supporting). **Edward E. Carey:** Conceptualization (supporting); Visualization (supporting); Writing‐review & editing (supporting). **Maria Isabela Andrade:** Supervision (equal); Writing‐review & editing (supporting). **Suzanne M Johanningsmeier:** Formal analysis (equal); Validation (equal); Writing‐review & editing (supporting). **Lora Forsythe:** Methodology (supporting); Writing‐review & editing (equal). **Genevieve Fliedel:** Methodology (supporting); Writing‐review & editing (supporting). **Tawanda Muzhingi:** Conceptualization (supporting); Funding acquisition (supporting); Project administration (lead); Supervision (supporting); Writing‐review & editing (supporting).

### Peer review

The peer review history for this article is available at https://publons.com/publon/10.1111/ijfs.14792.

## Data Availability

The data that support the findings of this study are available on request from the corresponding author. The data are not publicly available due to privacy or ethical restrictions.
